# Supplementary material for: Propensity score methods for comparative-effectiveness analysis: A case study of direct oral anticoagulants in the atrial fibrillation population
Source: PLoS One. 2022 Jan 24;17(1):e0262293. doi: 10.1371/journal.pone.0262293 (PMC8786176; doi:10.1371/journal.pone.0262293)
Supplement: S1 Table — ICD:10 = International Statistical Classification of Diseases and Related Health Problems 10th Revision, OPCS-4 = Classification of Interventions and Procedures, BNF = British national formulary, NRS = national records of Scotland. (PDF) [file pone.0262293.s001.pdf]

Outcome	Diagnostic, procedure and drug codes
Stroke-all	ICD-10 for Ischaemic stroke: I63, I64, G46.3-G46.7<br>OPCS-4 for Ischaemic stroke: U54.3<br>ICD-10 for Haemorrhagic stroke: I60-61
Major bleeding	Including Haemorrhagic stroke, GI bleeding plus<br>ICD-10 codes for Other major bleeds: D62, H11.3, H35.6, H43.1, I62, J94.2, N02, R04, R31, R58, N95.0
Mortality (all-cause)	Death identified from NRS
