# Supplementary material for: Propensity score methods for comparative-effectiveness analysis: A case study of direct oral anticoagulants in the atrial fibrillation population
Source: PLoS One. 2022 Jan 24;17(1):e0262293. doi: 10.1371/journal.pone.0262293 (PMC8786176; doi:10.1371/journal.pone.0262293)
Supplement: S2 Table — ICD:10 = International Statistical Classification of Diseases and Related Health Problems 10th Revision, OPCS-4 = Classification of Interventions and Procedures, BNF = British national formulary, VTE = venous thromboembolism. (PDF) [file pone.0262293.s002.pdf]

Outcome	Diagnostic, procedure, and drug codes
**Inclusion criteria**	
Atrial fibrillation	ICD-10: I48
Oral anticoagulant	BNF: 02.08.02
**Exclusion criteria**	
Mitral stenosis, valvular disease or heart valve replacement	ICD-10: I05 – I08, I34 – I37, Q22, Q23, Z95.2 – Z95.4<br>OPCS-4: K02.3, K25.3, K25.4, K25.8, K25.9, K26.3, K26.4, K29.1 – K29.4, K30.1, K31.1
VTE	ICD-10: I26, I63.6, I67.6, I80.1-I80.9, I81, I82.2- I82.9
