# Supplementary material for: Propensity score methods for comparative-effectiveness analysis: A case study of direct oral anticoagulants in the atrial fibrillation population
Source: PLoS One. 2022 Jan 24;17(1):e0262293. doi: 10.1371/journal.pone.0262293 (PMC8786176; doi:10.1371/journal.pone.0262293)
Supplement: Attachment — Submitted filename: Response_to_Reviewers.docx [file pone.0262293.s003.docx]

**Reply to reviewers’ comments for the manuscript titled “Propensity score methods for comparative-effectiveness analysis: a case study of direct oral anticoagulants in the atrial fibrillation population” (PONE-D-21-15771)**

We would like to thank both reviewers for their time and valuable comments. We have addressed these as follows.

**Reviewer#1: In the abstract it should be added how the models performed best, that is which outcomes were evaluated to assess performance of the model.**

**Response**: In the Abstract the following is already stated: “Model performance was assessed by standardised difference. Clinical outcomes and mortality were compared for all DOACs (including apixaban, dabigatran and rivaroxaban) versus warfarin”. The outcomes used in our model are now specified in the methods section of the abstract (line 41). However, the clinical outcomes where not used to assess model performance, but for indicating how the size of the treatment effect (reported in the form of Hazard Ratios) may change according to the PS method employed. For instance, with the IPTW-IPCW approach, the treatment effect tends to be larger, but still in line, compared to the treatment effect estimated with other PS methods. This is now stated in the results section of the Abstract (line 48 and 49).

**Reviewer#1: why patients in "dabigatran only" were assessed? and not rivaroxaban etc?**

**Response:** An explanation on why patients on “dabigatran only” were assessed is already provided in the manuscript; that is the smallest subgroup of DOACs users in Scotland compared to apixaban and rivaroxaban. However, this has now been made clearer (line 169).

**Reviewer#1: Methods: I would remove propensity score to mimic RCT, which is correct but not formally true.**

**Response**: The sentence referring to propensity score mimicking RCT has now been deleted (line 174).

**Reviewer#1: methods dealing with missing value should be added.**

**Response:** No methods for dealing with missing value were used. Given that missing data was <5%, no imputation method was used, a complete available case analysis was provided instead. This is now stated in the manuscript (line 194-196).

**Reviewer #2: Just one comment on the concept of the introduction of dabigatran in clinical practice. The use of dabigatran as opposed to warfarin is more linked to the more convenient management of the drug than to other reasons. This is what has happened in the recent years in the clinical practice, but the same also is seen for apixaban and rivaroxaban.**

**Response**: More on the “more convenient management” of DOACs versus warfarin has now been added to the methods section (line 110-13). That is, rapid onset of action, and the possibility of using fixed doses without requirement for routine anticoagulant monitoring.
